# Leucine-Rich Repeat Extension 7 Gene Confers Cotton Resistance to Verticillium Wilt

**DOI:** 10.3390/ijms27093852

**Published:** 2026-04-26

**Authors:** Xue Du, Yanfang Li, Wankui Gong, Zhen Wei, Qiankun Liu, Aiming Zhang, Yuting Ge, Yangyang Wei, Yuling Liu, Quanwei Lu, Xianghui Xiao, Pengtao Li, Juwu Gong, Renhai Peng

**Affiliations:** 1 School of Biotechnology and Food Engineering, Anyang Institute of Technology, Anyang 455000, China; duxue101199@163.com (X.D.); sybks10lyf25@163.com (Y.L.); liuthundering@163.com (Q.L.); m904780569y@163.com (A.Z.); geyuting2026@163.com (Y.G.); weiyangyang511@126.com (Y.W.); liuylay2012@163.com (Y.L.); daweianyang@163.com (Q.L.); xiaoxianghui4953@163.com (X.X.); 2 School of Life Sciences, Zhengzhou University, Zhengzhou 450001, China; 3 College of Agriculture, Tarim University, Alar 843300, China; 4 State Key Laboratory of Cotton Bio-Breeding and Integrated Utilization, Institute of Cotton Research of the Chinese Academy of Agricultural Sciences, Anyang 455000, China; gongwankui@caas.cn (W.G.); gongjuwu@caas.cn (J.G.)

**Keywords:** LRX gene family, *Gossypium hirsutum*, bioinformatics, *Verticillium dahliae*, VIGS

## Abstract

Leucine-rich repeat extensins (LRXs) are essential regulators of plant development, cell wall integrity, and stress responses. However, genome-wide LRX studies in cotton are limited. Analysis of four *Gossypium* species identified 29, 28, 16, and 16 LRX genes in *G*. *hirsutum*, *G*. *barbadense*, *G*. *arboreum*, and *G*. *raimondii*, respectively. Phylogenetic analysis resolved these 89 genes into four subfamilies (I–IV). Structural annotation revealed that cotton LRX family members exhibit conserved domain architectures. This finding was corroborated by motif analysis, which revealed notable conservation in the motif compositions of most cotton LRX proteins, suggesting functional conservation across evolutionary lineages. Distinct spatiotemporal expression patterns were uncovered between *G. hirsutum* and *G. barbadense*. Prolonged exposure to extreme temperatures induced widespread down-regulation of most *GhLRX* genes, whereas genes in subgroup IV were significantly up-regulated under salt and drought stress conditions, respectively. Notably, *GhLRX7* showed a more proactive responding profile to *Verticillium* wilt (VW) infection, which was therefore selected for functional validation employing virus-induced gene silencing in the cotton cultivars MBI9626 and CCRI36. Phenotypic analysis of silenced plants revealed exacerbated disease symptoms compared to wild-type controls, providing direct evidence implicating *GhLRX7* as a key contributor to defense against VW.

## 1. Introduction

The plant cell wall, a distinct and dynamically regulated scaffolding structure, is a defining feature distinguishing plant cells from animal cells. As a multifunctional barrier, it not only dictates the architecture of cells but also safeguards against biotic threats and abiotic stresses [1,2]. Biochemically, this complex structure is dominated by polysaccharides including cellulose, hemicellulose, and pectin. Although proteins comprise a minor component in the cell wall, they play critical functional roles [3]. These cell wall-associated proteins are pivotal in modulating intercellular signaling in plant development, and stress-responsive pathways [4,5]. Among them, extensins (EXTs), characterized by their conserved Ser-Pro2-n repeat motifs, are fundamental structural elements of cell wall proteins [6,7]. Particularly, EXTs have been reported to promote cell wall loosening and expansion, thereby driving cell enlargement and coordinating the growth of the entire cell wall [8,9]. A specific subgroup of the EXT family is the leucine-rich repeat extensins (LRXs), which are characterized by highly conserved LRR domains and EXT domains [4]. The LRR domain is responsible for a wide range of molecular recognition events, spanning from small signaling peptides to large structural proteins. Conversely, the EXT domain is involved in determining subcellular localization and regulating protein stability [10,11]. Phylogenetic clustering analysis of the 11 LRX members in Arabidopsis, based on their expression profiles, distinguished them into two classes: Class I (*AtLRX1–7*), which are enriched in vegetative tissues, and Class II (*AtLRX8–11*), which are pollen-specific [4,12]. From a functional perspective, Class I AtLRX proteins are likely essential for cell wall biogenesis of root hairs [13,14], while Class II members ensure the structural integrity of growing pollen tubes [15]. These functional divergences highlight the significant contribution of LRX proteins to tissue-specific cell wall remodeling and developmental processes.

Cotton is grown in over 80 countries worldwide, with approximately 30 of them growing cotton as their major commercial crop [16]. It provides approximately 35% of global natural fiber for the textile sector, while is also an important source of edible oil and livestock feed [17]. It was estimated that the global cotton output reached 25.343 million tonnes during the 2022–2023 season [18], led by China (6.1 million tonnes), India (5.99 M tonnes), the United States (3.06 M tonnes), Brazil (2.83 M tonnes), and Pakistan (0.98 M tonnes), with other top cotton producers. Different cotton species display substantial divergence in morphology, including distinct leaf shapes, fiber qualities, and a diverse range of plant architectures, from perennial wild trees and shrubs to annual cultivated herbs [19]. Cotton yield and fiber quality are affected by a range of stresses, including abiotic stresses (drought, salinity, and temperature) and biotic agents (pests, nematodes, and microbial pathogens) [20,21], in particular, the *Verticillium* wilt (VW) of cotton, a highly destructive and pathogenic disease, which is widespread when environmental factors are favorable. In China, the losses caused by VW represented 32.49% of the total losses due to all diseases in 2021. Over the past five years, the recovery rate of losses caused by VW has continued to be low, compared with other cotton diseases and insect pests [22]. In recent years, the severity of VW has been escalated, mainly driven by the combined effects of climate change and modern agricultural practices, specifically the long-term continuous monocropping in global cotton-growing regions and the continuous introduction of new varieties/hybrids [23].

*V*. *dahliae* is an important difficult-to-control soil-borne vascular pathogen, primarily due to its unique life cycle. It survives in the soil for several years as microsclerotia, a dormant stage that germinates upon stimulation by root exudates of a susceptible host. The pathogen then invades through root tips or wounds, colonizes the xylem vessels, and spreads upward with the transpiration stream, eventually blocks the vasculature and causes typical symptoms such as plant wilting, chlorosis, and vascular discoloration [24]. Disease development is accelerated at temperatures below 20 °C, and following host death, *V. dahliae* produces new microsclerotia in the decaying tissues, which are retained in the soil, thereby completing its infection cycle [24,25]. This resilient life cycle renders chemical and cultural control measures largely ineffective, making the breeding of resistant cultivars the most fundamental and sustainable management strategy [24]. The resistance of cotton plant to VW entails a sophisticated interplay of molecular events, including architectural reinforcement of cell walls, hormonal signaling cascades, and the triggering of pathogen suppression pathways. These mechanisms specifically encompass the biosynthesis and deposition of structural reinforcing substances, the biosynthesis of antifungal metabolites, hormonal signaling cascades, and the maintenance of reactive oxygen species (ROS) homeostasis [26,27]. Through in-depth research, critical players in these pathways have been identified, including genes such as *GhPGIP1*, *GhPMEI3*, and *GhRFS6*, which enhance cell wall integrity via regulating pectin metabolism and thereby inhibiting the proliferation of *V. dahliae* [28,29,30]. Genes such as *GhLAC15* and *GhWRKY1* promote the accumulation of lignin, suberin, and cork within vascular tissues, forming a physical barrier to prevent pathogen colonization [31,32,33]. Furthermore, the *GhSNAT1* and *GhWRKY41* genes orchestrate the biosynthesis of antifungal metabolites, including flavonoids and gossypol, to suppress fungal proliferation [34,35]. Plant innate immunity primarily relies on two fundamental layers: pattern-triggered immunity (PTI) and effector-triggered immunity (ETI). These defensive layers are governed by distinct receptors, including receptor-like proteins (e.g., *Ve1*) and nucleotide-binding site-leucine-rich repeat (NBS-LRR) proteins such as GbRVd [36,37,38]. The jasmonic acid (JA) and salicylic acid (SA) signaling pathways also orchestrate the cotton immune network, playing pivotal roles in defense activation. Additionally, the genes *GhRbohD* and *GbNRX1*, which orchestrate ROS homeostasis, protect cotton from oxidative damage and potentiate its defense responses [17,39]. In recent years, several VW-resistant upland cotton cultivars, including Jinken 1775 [40] and Zhongmian EB005 [41], have been identified; however, the genetic mechanism of resistance of these cultivars remains largely elusive [42], and many cultivars exhibit trade-offs in agronomic traits, such as reduced yield or compromised fiber quality, which has limited their large-scale adoption [43]. Thus, uncovering novel resistance genes and their molecular mechanisms are of both theoretical and practical importance for cotton breeding.

Studies have established that several LRX family members are pivotal regulators of plant morphogenesis. For instance, in *Arabidopsis*, the members *AtLRX1* and *AtLRX2*, primarily expressed in the meristematic zone, are indispensable for root hair development. The single mutants of *lrx1* or *lrx2* exhibited striking phenotypic disparities in root hair development: the root hairs of *lrx1* displayed severely distorted forms, whereas those of *lrx2* showed indistinguishable morphological differences from wild-type plants [13]. Strikingly, the *lrx1 lrx2* double mutants displayed more pronounced enhanced root hair defects than the *lrx1* single mutants, revealing functional redundancy between *LRX1* and *LRX2* [14]. Single mutants of *AtLRX3*, *AtLRX4*, or *AtLRX5* displayed no obvious phenotypes, suggesting functional redundancy of these LRX members, whereas the triple mutants manifested pleiotropic defects, including aberrant development, disrupted cellular and vacuolar morphogenesis, and elevated anthocyanin accumulation [44,45,46]. Furthermore, some studies demonstrated that a specific subset of LRX proteins (AtLRX8-11), which are expressed exclusively in pollen, is essential for the normal development and function of pollen of *Arabidopsis*, as key regulators of pollen germination and pollen tube elongation. The quadruple mutants of *lrx8*-*lrx11* exhibited severe reproductive defects, including decreased male fertility, reduced seed setting rate, and arrested pollen tube growth, which directly demonstrates the indispensable role of these LRX proteins for successful reproduction [12,47]. As an integral regulator, LRX is also involved in coordinating plant adaptive responses to stress. Lignin, a fundamental component of the plant cell wall, constitutes the primary physical barrier against pathogen invasion [48,49,50]. The cotton histone deacetylase gene *GhHDA5* is inhibited by *V. dahliae*. Silencing of *GhHDA5* leads to increased enrichment of H3K9K14ac at the promoters of the lignin biosynthetic genes *GhF5H* and *Gh4CL3*, thereby promoting gene transcription and increasing lignin content, particularly the content of S-type monomers [51]. After a genome-wide association study (GWAS) on the lignin response to VW (LRVW) values in cotton stem, candidate intervals associated with LRVW values were identified, among which LRX was included. Since the LRVW of the parental plants is significantly negatively correlated with the VW severity index, it is hypothesized that LRX contributes to cotton resistance to VW via regulating cell wall development and lignin synthesis [52].

Despite these findings, the systematic characterization of the LRX genes in cotton and their functional significance in regulating resistance to VW remains unexplored. The genus *Gossypium* encompasses 45 diploid (2*n* = 2x = 26) and seven tetraploid (2*n* = 4x = 52) species [53]. The progenitors of diploid *G. raimondii* (D genome) and *G. arboreum* (A genome) are supposed to generate the progenitors of the allotetraploid AD-genome species, which includes the two major cultivated species, *G. hirsutum* and *G. barbadense* [54]. Representing two different ploidy levels and the major genome types of the genus [53], this study aimed to comprehensively identify and characterize the LRX gene family in these four *Gossypium* species. The study included genome-wide analysis, the spatiotemporal expression patterns of LRX genes under various stress conditions, and the functional role of the candidate gene *GhLRX7* in cotton defense against *V. dahliae* using virus-induced gene silencing (VIGS) strategies. This study provides novel insights into the critical role of LRX genes in cotton resistance to VW, thereby establishing a foundation for future molecular breeding strategies aiming at enhancing disease resistance in cotton. Its functions have been documented in *A*. *thaliana* [55], tomato, maize, and rice [55,56,57], as well as loblolly pine and Norwegian spruce [58]. However, studying the role of LRX in regulating VW tolerance in plants, especially in cotton, is still in its early stages. A systematic functional characterization of this gene family in cotton still remains elusive. To elucidate the functions of LRXs in cotton, a genome-wide identification of these genes was performed. The genome-wide screening identified 89 LRX genes across the four representative *Gossypium* species, namely *G*. *hirsutum*, *G*. *barbadense*, *G*. *arboreum*, and *G*. *raimondii*. Comprehensive bioinformatics analyses were performed to characterize LRX genes in their physicochemical properties, conserved motifs, chromosomal locations, phylogenetic relationships, and spatiotemporal expression patterns. The *G. hirsutum* cultivar CCRI36, characterized by high yield and broad adaptability, was used as susceptible and MBI9626-resistant lines in the study. QRT-PCR was employed to quantify *GhLRX7* expression profiles in response to *V. dahliae* infection. The function of *GhLRX7* was specifically explored using VIGS. The results revealed that expression of *GhLRX7* was significantly induced by the pathogen infection while the *GhLRX7*-silenced plants exhibited pronounced leaf yellowing and wilting. These findings reveal the crucial role of the LRX gene family in cotton response to VW infection, providing new insights for further unraveling the defense mechanisms of cotton against VW.

## 2. Results

### 2.1. Genomic Identification and Bioinformatic Profiling of the Cotton LRX Gene Family

A genome-wide screening for LRX genes via BLASTP (2.17.0) against the reference genomes of *G*. *hirsutum*, *G*. *barbadense*, *G*. *arboreum*, and *G*. *raimondii* yielded 29, 28, 16, and 16 LRXs respectively. Table S1 shows the chromosomal locations, protein lengths, relative molecular masses, isoelectric points, Instability Index, Aliphatic Index and protein hydrophilicity of the 89 LRXs, which were named following their species and chromosomal positions: *GhLRX1*-*GhLRX29*, *GbLRX1*-*GbLRX28*, *GaLRX1*-*GaLRX16*, and *GrLRX1-GrLRX16*. The *LRXs* encoded amino acid sequences spanning 277 to 762 residues, with a predicted molecular weight, isoelectric point, Instability Index, and Aliphatic Index ranging 30.66–81.93 kDa, 4.6–9.85, 29.43–103.49, and 51.06–107.65, respectively. Protein hydrophobicity analysis showed that 58 LRXs are hydrophilic and 31 hydrophobic (Table S1).

### 2.2. Evolutionary Relationships Within the Cotton LRX Gene Family

In *G. hirsutum*, the *GhLRXs* are distributed across 17 chromosomes, with each chromosome harboring 1–3 of them. In *G*. *barbadense*, the *GbLRXs* are also distributed across 17 chromosomes, with chromosome A01 harboring the highest number of four genes. In *G*. *arboreum,* the *GaLRXs* are distributed across nine chromosomes, with chromosome one harboring four *GaLRXs*. In *G*. *raimondii*, the *GrLRXs* are distributed across 10 chromosomes (Figure S1a). The systematic arrangement of LRX members across chromosomal loci in these *Gossypium species* reveals non-random organizational principles, which establishes a framework for subsequent functional investigations of this gene family.

Phylogenetic analysis of LRX genes from *Arabidopsis*, *Zea mays*, and *Oryza sativa*, and four *Gossypium* species classified them into four major subfamilies, with cotton LRX genes evenly distributed among these subfamilies (Figure S1b; Supplementary File S1). Subfamily I was the largest, comprising 41 members, which accounted for approximately one-third of the total. Subfamily II contained 39 members, Subfamily III and IV each contained 15 members. A comparison of LRX members revealed that the diploid progenitors, A genome of *G. arboreum* and D genome of *G. raimondii*, contained approximately half the number of genes of the allotetraploid species, *G. hirsutum* and *G. barbadense*, which are consistent with their ploidy levels. Within the same subfamily, homologous genes in diploid or tetraploid species maintained a correspondent chromosomal distribution. This observed strong phylogenetic affinity between *G. hirsutum* (Gh) and *G. barbadense* (Gb) was attributed to their shared tetraploid ancestry.

### 2.3. Collinearity of LRXs in Cotton

**Figure 1:**
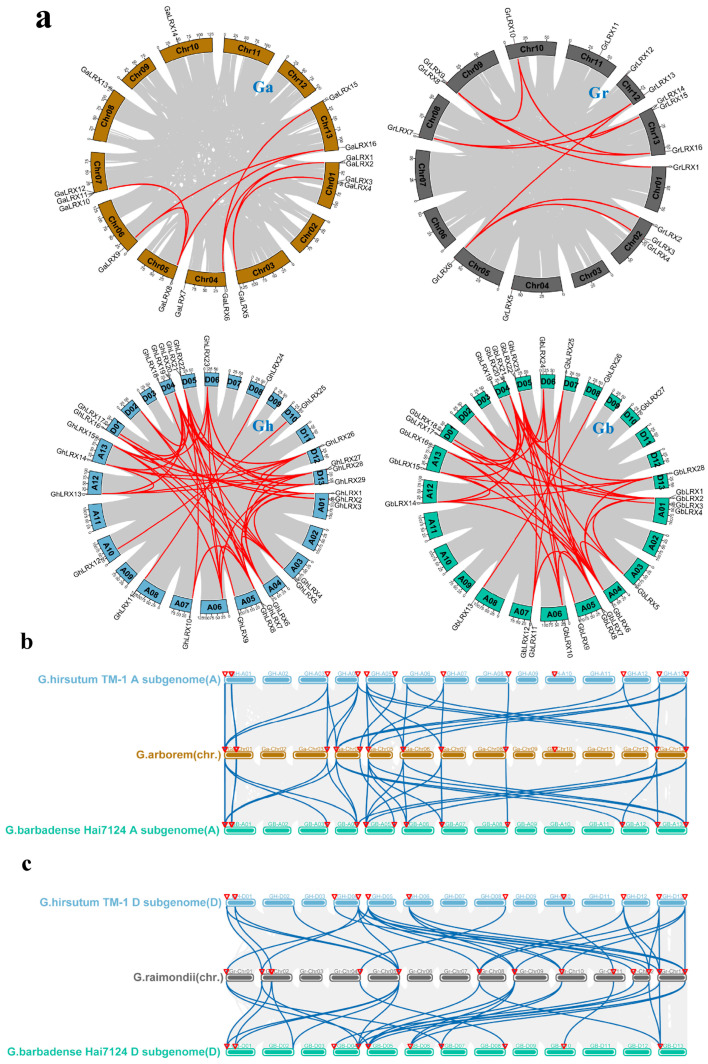
Visualization of collinear relationships among LRX genes. (**a**) Intraspecific synteny maps for the four analyzed *Gossypium* species. (**b**) Collinearity analysis between A genome of *G. arboreum* and at subgenomes of *G. hirsutum* and *G. barbadense*. (**c**) Collinearity analysis between D genome of *G. raimondii* and Dt subgenomes of *G. hirsutum* and *G. barbadense*. Red lines represent intraspecific collinear blocks within each of the four *Gossypium* species, blue lines represent interspecific collinear blocks between species, and the red triangles represent the positions of LRX genes on the chromosomes.

The emergence of gene families is primarily driven by large-scale genomic amplification events, including duplications of whole-genome, chromosomal tandem or segment. Intraspecific synteny analysis of LRXs of the four *Gossypium* species obtained eight, 10, 42, and 50 pairs of duplicate genes in Gr-, Ga-, Gb-, and Gh-genomes, respectively (Figure 1a). Interspecific synteny analysis of LRXs revealed a conserved collinear relationship among the four *Gossypium* species. Orthologous relationships were observed between the LRX members on the A_t_/D_t_ subgenomes of *G. hirsutum* and *G. barbadense* and those at the corresponding positions on the A/D genomes of *G. arboreum*/*G. raimondii* (Figure 1b,c). A systematic synteny analysis uncovered extensive homologous relationships within the LRX family across the four cotton species. Specifically, the diploid Ga-A genome showed 26 and 27 syntenic sites with the tetraploid Gh-A_t_ and Gb-A_t_ subgenomes, respectively, while the diploid Gr-D genome exhibited 28 and 25 syntenic sites with the tetraploid Gh-D_t_ and Gb-D_t_ subgenomes. Additionally, in A-A_t_ genomes, there were more homologous LRX pairs on chromosomes one, five, and 13 between the diploid Ga-A genome and the tetraploid Gh-A_t_ and Gb-A_t_ subgenomes than the other chromosomes. However, in D-D_t_ genomes, there were more homologous LRX pairs on chromosomes one, four, and 13 between the diploid Gr-D genome and the tetraploid Gh-D_t_ and Gb-D_t_ subgenomes than the other chromosomes. These results indicate that the LRX family exhibits high conservation among the four major cotton species.

### 2.4. Conserved Motif and Cis-Acting Element (CAE) Analyses of LRX in Cotton

Conserved motif analysis is a complementary strategy to characterize LRX function (Figure S1c; Supplementary File S2). The LRX amino acid sequences have up to 10 conserved motifs, named as motif one to 10. In *G*. *hirsutum*, all 29 *GhLRXs* possess motifs one to four, but *GhLRX21* and *GhLRX28* lack motif 10, *GhLRX9* and *GhLRX18* lack motif nine, and *GhLRX26* lacks motif five. In *G*. *barbadense*, all 28 *GbLRXs* possess motifs one, three, and four, *GbLRX9* and *GbLRX19* lack motif nine, and *GbLRX14* and *GbLRX15* lack motif 10. In *G*. *arboreum*, all 16 *LRXs* contain motifs 1–4 and six, but *GaLRX7* lacks motif nine, *GaLRX10* lacks motif five, and *GaLRX14* lacks motif 10. In *G*. *raimondii*, all *GrLRXs* possess motifs 1–5, *GrLRX12* lacks motif nine, and *GrLRX14* and *GrLRX15* lack motif 10. The conserved motif sequences in cotton LRX family proteins are highly similar, suggesting strong conservation within the LRX family in *Gossypium*.

**Figure 2:**
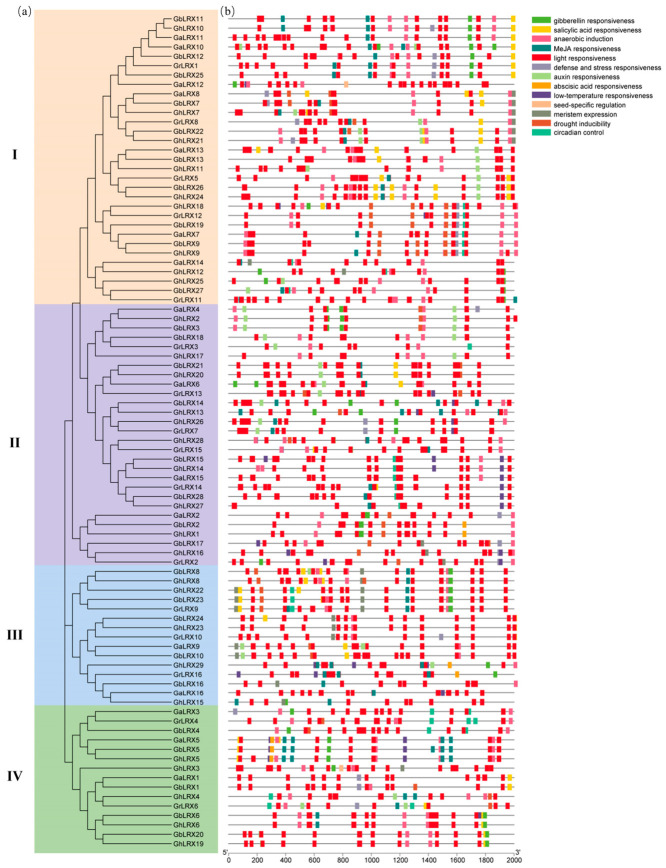
Promoter cis-acting elements of cotton LRX genes. (**a**) An evolutionary tree of all the cotton *LRX* genes. The colors of yellow, purple, blue, and green represented the subgroups of I–IV, respectively. (**b**) The predicted cic-acting elements of all the cotton *LRX* genes.

The thirteen identified CAEs were classified into three principal categories: developmental processes, hormonal signaling pathways, and abiotic stress adaptation (Figure 2). The CAEs of developmental processes include those of anaerobic induction, meristem-specific activation, circadian control, and seed development; the CAEs of hormonal signaling pathways include those of auxin, abscisic acid, meiotic-jasmonic, salicylic acid, and gibberellin responsiveness; the CAEs of abiotic stress adaptation include those of light response, drought induction, cold response, defense, and stress response. Overall, the CAEs of light response were the most abundant ones in the promoter region of cotton LRXs, followed by the ones of anaerobic induction, of methyl jasmonate (MeJA) response, and of abscisic acid response. The abundance of light-responsive and MeJA-responsive elements suggests that LRX genes may integrate light and jasmonate signaling to regulate biotic and abiotic stress responses. This valuable information will facilitate the exploration and validation of potential functional roles for cotton LRX in biological processes.

### 2.5. Identification of Enriched Transcription Factor-Binding Motifs in LRX Promoters

To elucidate the potential transcriptional regulatory mechanisms of the LRX gene family, the 2000 bp promoter regions of 89 LRX genes from four cotton species were analyzed. A total of 186 known plant transcription factor-binding motifs were identified (Table S2). The analysis revealed that the top 20 significantly enriched motifs corresponded to 10 distinct transcription factor families. The most significantly enriched motifs belong to the Dof (DNA binding with one finger) family, in which *CDF5* (Cycling Dof Factor 5) exhibited the highest significance. *CDF5* was identified to bind two motifs in the target promoter region with occurrence rates of 49.44% and 55.06%, while in the control sequences, the *CDF5* only exhibited a binding rate of 9.08% and 15.45% to the two motifs, respectively. Other significantly enriched Dof family members include *DOF3.6*, *DOF1.7*, *DOF1.5*, *DOF21*, and *DOF5.8*. In addition to Dof family members, motifs for *TSO1*, *ZHD6*, *AHL13*, and *TCX6* were also significantly enriched. Notably, motifs for *MYB83* and *MYB30*, two transcription factors known as key regulators of secondary cell wall biosynthesis in plants, were also significantly enriched. Other enriched transcription factors included *NAC076*, which is associated with vascular development, and *RIN*, a ripening regulator. Collectively, these results demonstrate that LRX gene promoters are significantly enriched in the binding sites of Dof transcription factors, particularly *CDF5*, as well as other regulatory factors such as MYB and NAC. These findings suggest that LRX genes may potentially participate in various biological processes including cell wall development and stress responses.

### 2.6. Analysis of Protein Interactions and Functional Enrichment for the GhLRX Gene

**Figure 3:**
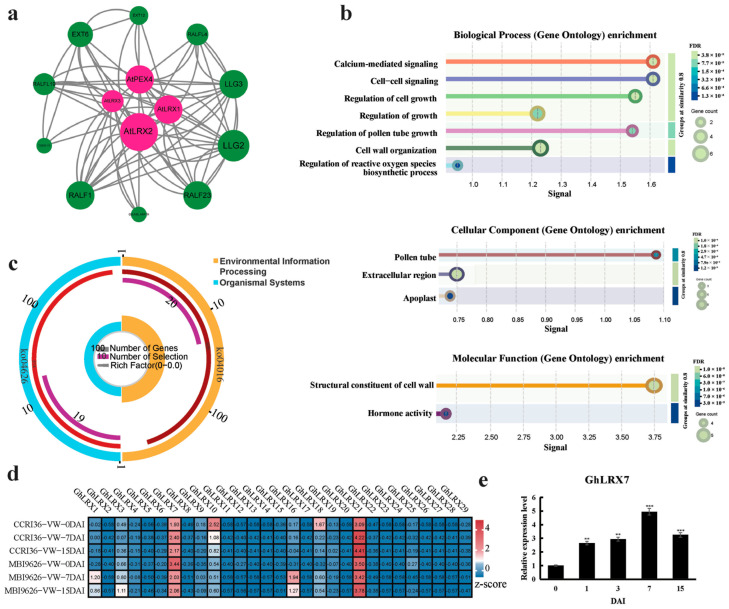
Interaction network, functional enrichment, and *Verticillium* wilt response of the GhLRX protein family. (**a**) Predicted *GhLRX* interactome from the STRING database. (**b**) GO enrichment analysis based on the STRING database. (**c**) KEGG pathways of the *GhLRX* genes. (**d**) Expression profile of *GhLRX* under *Verticillium dahliae* stress. (**e**) Relative expression levels of *GhLRX7* from 0 to 15 DAI under *V. dahliae* stress. Data are presented as means ± SD (*n* = 3). Asterisks indicate statistically significant differences compared with the control (0 h) (** *p* < 0.01, *** *p* < 0.001; *t*-test).

*G. hirsutum* accounts for over 90% of global cotton fiber production; therefore, the *G. hirsutum* lines were used for further qRT-PCR and VIGS-based functional validation in this study. Homology alignment of the LRX proteins between this species and *A*. *thaliana* revealed the existence of corresponding homologous relationships between these LRX proteins across the two plant species. The result showed that an LRX in *Arabidopsis* has at least one and at most twenty homologs in *G*. *hirsutum* (Table S3). Analysis of the protein interaction network revealed that *AtLRX2*, *AtPEX4*, *AtLRX1*, *LLG2*, *LLG3*, *RALF23*, and *RALF1* possess a high number of edges in the network (Figure 3a; Table S4), indicating their elevated centrality therein. These critical hubs imply their involvement in orchestrating a broader spectrum of biological processes in plants, including growth and development, cell wall assembly, signal transduction, and defense processes. In GO enrichment analysis, the most significant enriched terms in biological processes were calcium-mediated signaling (GO:0019722), cell–cell signaling (GO:0007267), regulation of cell growth (GO:0001558) and pollen tube growth (GO:0080092), and cell wall organization (GO:0071555); the terms in cellular components were pollen tube (GO:0090406), extracellular region (GO:000576), and apoplast (GO:0048046); the terms in molecular function were structural constituent of cell wall (GO:0005199) and hormone activity (GO:0005179) (Figure 3b). GO enrichment analysis reveals that the *GhLRX* gene family exhibits high specificity and functional consistency, primarily participating in the dynamic regulation of the cell wall and intercellular signal transduction processes. Analysis of KEGG pathways to further investigate the potential functions of these GhLRX proteins revealed that only two KEGG pathways, signal transduction (ko04016) and environmental adaptation (ko04626), were enriched by 20 and 19 *GhLRX* genes respectively (Figure 3c; Table S5). This suggests that the *GhLRX* gene family is primarily involved in the perception and transduction of environmental signals during plant adaptation to abiotic stresses, while during plant responses to biotic stresses, they may indirectly regulate the basal physical barrier defenses and immune signaling pathways in plants via detecting alterations in cell wall integrity.

### 2.7. Expression Profiling of LRX Genes in Response to V. dahliae Challenge

Systematical analysis of the expression profiles of 29 *GhLRX* genes based on published transcriptomic data of *G*. *hirsutum* revealed that *GhLRX* genes have complex and diverse expression profiles across various tissues—including root, stem, leaf, petal, receptacle, sepal, epicalyx, anther, and pistil (Figure S2a). These expression characteristics were also observed during cotton ovule and fiber development (Figure S2b). Under various abiotic stress conditions, the *GhLRX* genes had various responses in expression profiles (Figure S2c). In contrast, under VW stress, it was observed that *GhLRX7* and *GhLRX21* had significant expression responses with *GhLRX7* showing higher expression in the VW-tolerant line MBI9626 than in the VW-susceptible line CCRI36 (Figure 3d). Expression verification via qRT-PCR analysis further validated the transcriptomic data and highlighted the unique expression pattern of *GhLRX7* in MBI9626 (Figure 3e). The expression induction suggests that *GhLRX7* likely acts as a key inducible defense gene in cotton’s response to pathogen invasion, with its expression closely correlated with infection progression.

### 2.8. Functional Verification of GhLRX7 in Regulating Cotton Resistance to VW via VIGS

**Figure 4:**
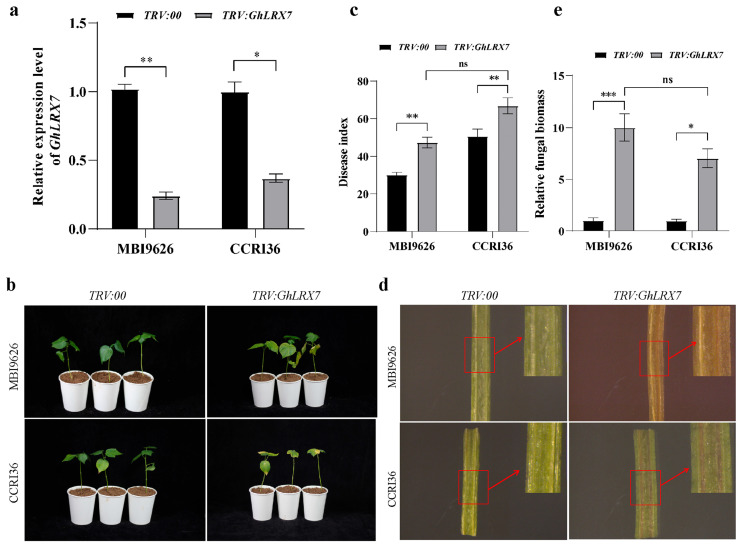
VIGS-based functional characterization of *GhLRX7* in VW resistance. (**a**) Validation of *GhLRX7* knockdown efficiency in cotton cultivars MBI9626 and CCRI36. (**b**) Enhanced disease susceptibility in *TRV:GhLRX7* plants, displaying more severe symptoms than TRV:00 controls at 25 DAI. (**c**) Disease severity index quantification confirming significantly higher susceptibility upon *GhLRX7* silencing. (**d**) Comparative stem vascular morphology between control and *GhLRX7*-silenced plants (scale bar = 0.2 cm). (**e**) Increased fungal biomass of *V. dahliae* in *TRV:GhLRX7* plants, indicating compromised pathogen restriction. Data are presented as means ± SD (n = 3). Asterisks indicate statistically significant differences (* *p* < 0.05, ** *p* < 0.01, *** *p* < 0.001; *t*-test).

In the functional verification of *GhLRX7* in cotton resistance to VW via the VIGS experiment, *GhPDS*-silenced plants exhibited a typical photobleaching phenotype at 14 DAI, confirming the functionality of the VIGS system. In contrast, plants infiltrated with the empty *TRV2* vector (negative control) showed no obvious phenotypic alterations (Figure S3). Knockout efficiency of *GhLRX7* confirmed via qRT-PCR showed that the mRNA levels of *TRV:GhLRX7* plants of MBI9626 and CCRI36 decreased by approximately 75% and 80%, respectively (*p* < 0.01) (Figure 4a), indicating a successful knockout of *GhLRX7* in the VIGS plants. Disease assessment performed at 25 DAI revealed that *TRV:GhLRX7* plants exhibited significantly more severe disease symptoms including severe leaf wilting, chlorosis, and necrosis (Figure 4b). The VIGS plants of both MBI9626 and CCRI36 exhibited a significantly higher disease index (DI) than the *TRV:00* control plants, indicating a significantly reduced resistance to VW in the former (Figure 4c). Cross sections of stems showed that the vascular tissues of VIGS plants exhibited a large area of deeper browning and necrosis than the *TRV:00* control plants (Figure 4d) and their vascular tissues also accumulated a significantly higher fungal biomass compared to the *TRV:00* control plants (Figure 4e). Collectively, these results establish *GhLRX7* as a pivotal positive regulator of cotton defense against VW.

## 3. Discussion

LRX proteins are chimerical extensins localized in the cell wall, playing key roles in plant morphogenesis and stress response [44,58,59,60]. The LRX gene family classifications have been established in model plants and crops, including *Arabidopsis* [55], tomato [56], maize [57], rice [55], as well as coniferous species of loblolly pine and Norwegian spruce [58]. LRXs in higher plants have evolutionarily diverged into two functionally specialized categories: those exhibiting predominant expression in vegetative organs, and those exhibiting specific activities in male reproductive tissues including pollen grains and pollen tubes [4].

Prior to this study, there is no genome-wide identification analysis of the LRX gene family performed on cotton species. Therefore, in this study, genome-wide identification of the LRX gene family was performed in four specific cotton species, *G*. *hirsutum*, *G*. *barbadense*, *G*. *arboreum* and *G*. *raimondii*, and a total of 89 *LRXs* were identified. The results showed that the LRX family has undergone expansion in all four cotton species and their numbers exceed the numbers documented in *Arabidopsis*, rice, maize, grapevine, and Norway spruce [55,56,57,58,61]. In addition, comprehensive characterization analysis was carried out on these family genes, including physicochemical properties, conserved motifs, chromosomal distribution, and phylogenetic history. This differs from the conventional classification system in previously reported plant species [62]. Such classification variations highlight the role of lineage-specific features in shaping the evolutionary trajectories of multigene families [63,64]. The architectural features of different gene families can illuminate their phylogenetic history and diversification mechanisms [65,66], while their conserved motifs can be used to predict the functions of their encoded proteins [67,68]. The functions of LRXs in *Arabidopsis* have been characterized, showing that *AtLRXs* function as key regulators coordinating multiple processes of plant morphogenesis and organ development, and different subfamilies exhibit distinct functions. Among the AtLRXs localized in vegetative organs, AtLRX1 and AtLRX2 are essentially required for normal cell wall biogenesis during root hair formation [13,14]. AtLRX3, AtLRX4, and AtLRX5 are also involved in cell wall biogenesis. The *lrx3/lrx4/lrx5* triple-mutant *Arabidopsis* plants displayed retarded growth and development with altered leaf architecture, impaired epidermal integrity, abnormal cellular morphogenesis and vacuolar expansion, as well as enhanced anthocyanin accumulation [44,45,46]. The pollen-expressed AtLRX8, AtLRX9, AtLRX10 and AtLRX11 cooperatively function to maintain structural integrity of pollen tubes and ensure successful pollen germination [4,12].

In the current study, evolutionary and sequence analyses support the division of cotton LRX proteins into four distinct evolutionary clades (Figure S1b). The variation in LRX motif arrangement across clades (Figure S1c) suggests that the functional roles of different cotton LRX groups may have undergone evolutionary shifts to facilitate cotton biological adaptation to environment changes. Specifically, the conserved motifs of LRXs in subfamilies III and IV share higher similarity with each other than that of group I and II (Figure S1c). Relevant studies on AtLRX proteins in *Arabidopsis* have demonstrated that conserved domain architecture and gene organization underlie parallel biological functions [33,55]. Based on these findings, it is inferred that LRX proteins in subfamilies III to IV are more likely to possess similar functional properties. Spatiotemporal expression patterns are valuable indicators for gene function annotation [69,70].

In this study, it was observed that the promoter regions of cotton LRX genes are rich in multiple CAEs, including light-responsive, MeJA-responsive, and ABA-responsive elements. The synergistic regulation between light and jasmonate signaling plays an important role in plant immunity [71]. Studies have shown that light not only influences plant metabolism through photosynthesis but also affects plant defense responses against pathogens by modulating the jasmonic acid signaling pathways [72]. Therefore, the co-enrichment of light-responsive and MeJA-responsive elements in the promoters of LRX genes suggests that these genes may act as crosstalk nodes integrating light and jasmonate signaling pathways, coordinating environmental and hormonal signals to regulate cotton defense against *V. dahliae*. In addition, the enrichment of ABA-responsive elements also warrants attention. ABA typically functions as a negative regulator in plant stress responses and can attenuate plant resistance to biotrophic pathogens by suppressing the jasmonic acid signaling pathway [73,74]. The presence of ABA-responsive elements in LRX gene promoters implies that these genes may participate in ABA-mediated stress response pathways, although their specific functions in cotton resistance to VW remain to be further investigated. Collectively, the enrichment patterns of CAEs in the promoter regions provide valuable clues for deciphering the regulatory mechanisms of LRX genes in cotton VW resistance, and warrant further exploration of their upstream regulatory networks.

The expression patterns of cotton LRX genes exhibit unique species-specific characteristics. Notably, *GhLRX7* displays a strong induced response to VW infection, a feature that has not been reported for its *Arabidopsis* orthologs *AtLRX3/4/5*. Similarly, in grapevine, *VvLRX7* is significantly induced by salt stress, and its overexpression enhances salt tolerance in *Arabidopsis* [61]. In Brassica rapa, *BrLRX2* and *BrLRX6* are markedly up-regulated under salt stress [75]. Collectively, these findings suggest that the LRX gene family has undergone lineage-specific expansion and functional divergence across different plant species, and the species-specificity of their expression patterns may be closely associated with adaptive evolution to particular environmental stresses [55]. Therefore, caution should be exercised when extrapolating knowledge of LRX gene functions from model plants to crops such as cotton, taking into account potential functional differences arising from species-specificity. At present, systematic research data on the spatiotemporal expression patterns of cotton LRXs still remains limited. The current study analyzed the spatiotemporal expression patterns of LRXs across various organs and stress treatments using existing transcriptomic resources (Figure S2). The results indicate that these genes possess diverse functions during the processes of cotton development and environmental adaptation. Especially, the expression of *GhLRX7* was strongly induced under VW stress conditions (Figure 3d). RT-qPCR also confirmed its significant induction in response to VW infection (Figure 3e), indicating its regulatory role in cotton response to VW stress. Functional validation of *GhLRX7* via VIGS showed that the VIGS cotton plants exhibited more severe disease phenotypes. However, how *GhLRX7* regulates cotton plant responses to VW infection is still not elucidated. In this study, we demonstrated that *GhLRX7* functions as a positive regulator of cotton defense against VW, as its silencing led to significantly exacerbated disease symptoms. In *Arabidopsis*, the closest orthologs of *GhLRX7* are *AtLRX3*, *AtLRX4*, and *AtLRX5*. It has been reported that *AtLRX3/4/5* act as cell wall integrity sensors and regulate multiple plant hormone signaling pathways—including those of JA, SA, and ABA—through the *LRX3/4/5*-*RALF22/23*-FER module, thereby coordinating plant growth and salt stress responses [76]. When this module is impaired, the JA and SA pathways become hyperactivated, leading to growth inhibition; concurrently, ABA homeostasis is disrupted and reactive oxygen species accumulate excessively, inducing cell death [77]. Although VW and salt stress are distinct types of abiotic and biotic stresses, the function of *GhLRX7* in cotton resistance to VW shares similarities with that of *AtLRX3/4/5* in *Arabidopsis* salt tolerance—both act as positive regulators in stress responses and both are associated with JA signaling. This finding suggests that LRX-mediated cell wall integrity sensing mechanisms may be conserved in plant responses to different stresses [78]. Although a distinct stress environment usually incurs specific transcriptional variation at the individual gene level [79,80], recent studies have shown that plants tend to adopt the same response to cope with various stresses [81]. These studies imply that *GhLRX7* may regulate cotton plant’s response to VW attack in a similar mechanism as it does in cotton response to salt stress [76]. However, further studies are still needed to elucidate the regulating mechanism of *GhLRX7* in cotton response to VW attack. These findings collectively demonstrate that *GhLRX7* serves a critical function in cotton’s defense against VW and establish a foundation for subsequent investigations of LRX gene functions in cotton and other species.

## 4. Materials and Methods

### 4.1. The LRX Gene Family and the Physicochemical Properties of Their Encoded Proteins in Cotton

Publicly available genomic data of cotton and *A*. *thaliana*, including DNA and protein sequences with annotation files, were sourced from the CottonMD (https://yanglab.hzau.edu.cn/CottonMD) (accessed on 12 October 2024) and Ensembl Plants (https://plants.ensembl.org/index.html) (accessed on 12 October 2024) databases, respectively. The following genome assemblies used in this study include: *G. hirsutum* (TM-1_ZJU v2.1 [82]), *G. barbadense* (H7124_ZJU v1.1 [82]), *G. raimondii* (D5_HAU v1.0 [83]), *G. arboreum* (A2_CRI v1.0 [84]), *A. thaliana* (TAIR10 [85]), *Zea mays* (B73 RefGen_v5 [86]) and *Oryza sativa* (IRGSP-1.0 [87]). The previously reported *Arabidopsis* LRX protein sequences were used as queries, local BLASTP searches were performed against the four cotton genomes with the following parameters: E-value threshold of 1 × 10^−5^, identity ≥ 40%, BLOSUM62 scoring matrix, gap opening penalty of 11, and gap extension penalty of 1. All candidate proteins were further validated using the Hidden Markov Model (HMM) profile of the LRX domain (PF13855) from the Pfam database [88] via HMMER with an E-value threshold of 1 × 10^−5^. Protein domain annotation of LRX proteins was performed using NCBI Batch-CDD with default parameters (E-value ≤ 0.01, maximum number of hits = 500). The physicochemical properties of the proteins were evaluated using the online Expasy ProtParam tool (https://web.expasy.org/protparam/) (accessed on 15 October 2024) with default parameters [89].

### 4.2. Chromosome Mapping of the Cotton LRX Family

Chromosome mapping of the cotton LRX family was performed by employing the Gene Location Visualize function from GTF/GFF program in TBtools-II [90]. First, genome annotation files (GFF format) of the four *Gossypium* species were downloaded from the CottonMD database, and a list of LRX gene names identified in each species was compiled. Subsequently, the GFF annotation files and the gene name lists were used as input files for the TBtools program. Based on the gene position information in the GFF files, the program automatically extracted the start position, end position, and chromosome number of each LRX gene. Finally, the program generated chromosomal distribution maps, visually displaying the distribution of LRX genes on each chromosome, including the density distribution and specific localization of genes across the chromosomes.

### 4.3. Evolutionary Relationships and Phylogenetic Analysis of Cotton LRX Genes

Multiple sequence alignment of LRX protein sequences from the four *Gossypium* species was performed using the MUSCLE algorithm implemented in MEGA X [91]. The alignment was conducted with default parameters (gap opening penalty: −2.9, gap extension penalty: 0, protein weight matrix: GONNET). Following alignment, poorly aligned regions and positions with many gaps were removed to optimize data quality for subsequent tree construction. Subsequently, a phylogenetic tree was reconstructed using the Neighbor-Joining method with 1000 bootstrap replicates to assess branch support. All alignment and tree construction analyses were performed using MEGA X (10.2.6) software. The resulting tree file (nwk format) was further refined and finalized for layout visualization using the iTOL [92] online platform (https://itol.embl.de/) (accessed on 19 October 2024).

### 4.4. Architecture of Conserved Motifs in the Cotton LRX Family

Conserved motifs of the LRX protein sequences of four cotton species were identified through the online MEME suite (https://meme-suite.org/meme/tools/meme) (accessed on 22 October 2024). The analysis employed the following parameter configuration: classical mode, zero or one occurrence per sequence, maximum number of motifs set to 10, and motif length range of 6–50 residues. Subsequently, the TBtools software was employed to generate a graphical representation of the conserved protein motifs [90].

### 4.5. Analysis of Motif Enrichment

To identify potential transcription factor-binding motifs enriched in the promoter regions of LRX gene family members, AME using the AME tool implemented in the MEME Suite was performed. The 2000 bp upstream promoter sequences of 89 *LRX* genes from *G. hirsutum*, *G. barbadense*, *G. raimondii*, and *G. arboreum* were extracted. The JASPAR CORE 2024 Plants database was used as the reference motif database. Control sequences were generated by shuffling the input sequences while preserving dinucleotide (2-mer) frequencies. Sequence scoring was performed using the average odds score method, and the statistical significance of motif enrichment was assessed using Fisher’s exact test. The hit threshold for motif occurrences was set to 0.25 of the maximum log-odds score, and the E-value reporting threshold was set to 10.0. Motifs with an E-value < 0.05 were considered significantly enriched.

### 4.6. Collinearity Analysis of LRXs in Cotton

Intraspecific collinearity analysis for each of the four *Gossypium* species was performed using the One Step MCScanX program in Tbtools-II [90] with genome annotation files and protein sequence files as inputs. This program integrates BLASTP alignment and the MCScanX algorithm to identify syntenic blocks within each genome, with the BLASTP E-value threshold set to 1 × 10^−5^. Interspecific collinearity analysis was conducted using the Dual Synteny Plot for MCscanX program in TBtools for two species triplets: *G. hirsutum*, *G. arboreum*, and *G. raimondii*; and *G. barbadense*, *G. arboreum*, and *G. raimondii*. The resulting collinearity relationships were visualized as genomic synteny plots using TBtools [90].

### 4.7. Identification of CAEs in the Promoter Regions of Cotton LRX Genes

DNA sequences spanning 2000 base pairs upstream of the translation initiation site of the cotton LRX genes were obtained from CottonFGD (http://cottonfgd.net/) (accessed on 9 July 2025) for subsequent promoter analysis. The putative CAEs of LRXs were predicted using the PlantCARE database (http://bioinformatics.psb.ugent.be/webtools/plantcare/html/) (accessed on 9 July 2025) to scan the above obtained sequences. Visualization of predicted CAEs was performed via TBTools, where colored rectangles represent distinct CAEs grouped by shared evolutionary branches.

### 4.8. Analysis of Protein Interactions and Functional Enrichment for GhLRX Gene

Given the absence of protein data of cotton species recorded in the STRING database (http://string-db.org) (accessed on 12 July 2025), homology comparisons were first conducted among the sequences of 29 *GhLRXs* and 10 *AtLRXs*. The protein sequences of the latter were input as model proteins into the STRING database to construct protein–protein interaction networks and Gene Ontology (GO) enrichment. The GO database adopts a tripartite classification system, encompassing organismal processes, cellular architecture, and molecular operations. Functional annotation of KEGG pathways of the *GhLRX* genes was performed using the web-based OmicShare tool (https://www.omicshare.com/) (accessed on 14 July 2025) and the genome annotation dataset of *G*. *hirsutum* was obtained from the KEGG database (https://www.kegg.jp/) (accessed on 14 July 2025).

### 4.9. Expression Patterns in the LRX Family

Transcriptional profiles of cotton *GhLRX* genes were extracted from the Cotton Omics Database (http://cotton.zju.edu.cn/10.rnasearch.html)(accessed on 23 December 2024) in tab-separated text format, which directly provided FPKM values covering expression across diverse tissues (including root, stem, leaf, petal, torus, sepal, epicalyx, anther and pistil) and responses to abiotic stresses (extreme temperature, salt stress, and drought stress). Previously published transcriptomic data [93] was applied to analyze the host transcriptomic responses to *V*. *dahliae* infection, and these data were downloaded in SRA format. For transcriptomic dataset normalization, raw read counts were first filtered to remove genes with low expression (less than 10 counts in all samples). The remaining counts were then normalized to FPKM (Fragments Per Kilobase of transcript per Million mapped reads) values to account for library size and gene length differences. For heatmap visualization, row-wise Z-score normalization was performed on the FPKM values, centering the expression level of each gene across all samples to a mean of zero with a standard deviation of one. This Z-score transformation enhances the comparability of expression patterns across genes by scaling their relative expression fluctuations. Heatmaps were constructed using the Omicshare platform (https://www.omicshare.com/tools/) (accessed on 20 July 2025).

### 4.10. Assessment of Gene Expression Levels Under VW Stress Condition

Sterilized vermiculite and sandy soil were mixed in a 6:4 ratio with water and filled in disposable paper cups. Ten uniform and plump seeds of each cotton cultivar, MBI9626 and CCRI36, were sampled separately and soaked for 24 h, then sown in the paper cups. The cups were covered with plastic film and placed in greenhouse at 28 °C. Four days after being sown, the seedlings were combed and five seedlings with consistent growth were retained in each paper cup. *V. dahliae* strain V991 was activated via culturing it on solid potato dextrose agar (PDA) medium (containing streptomycin sulphate). Then, the V991 inoculum was cultured in liquid Czapek medium supplemented with streptomycin sulphate for approximately 5 days at constant shaking speed of 180 rpm and room temperature. The fungal culture was filtered through sterilized gauze, then its spore concentration was tested using a hemocytometer and calibrated to 1 × 10^7^ spores/mL using sterile water. When the seedlings reached one true-leaf stage, they were inoculated with 2 mL of the spore suspension using the root-wounding method by removing the bottom of the paper cups. Fresh leaf tissues were sampled at 0, 1, 3, 7, 15 days after inoculation (DAI).

Total RNA was extracted from the fresh leaf tissues. The RNA concentration and purity were assessed using a NanoDrop 2000 spectrophotometer (Thermo Fisher Scientific, Waltham, MA, USA). For each sample, 1 µg of total RNA was used for cDNA synthesis. Complementary DNA (cDNA) was synthesized using the HiScript III RT SuperMix for qPCR (+gDNA wiper) kit (Vazyme, Nanjing, China) in a 20 µL reaction volume following the manufacturer’s protocol. The resulting cDNA was fivefold-diluted with nuclease-free water to a final volume of 100 µL. Subsequently, 2 µL of the diluted cDNA (containing approximately 10 ng of cDNA) was used as the template per qPCR reaction. The qPCR reaction mixture (20 µL total volume) contained 10 µL of 2× ChamQ Universal SYBR qPCR Master Mix, 0.4 µL each of forward and reverse primers (10 µM), 2 µL of diluted cDNA, and nuclease-free water to reach the final volume. The thermal cycling program consisted of an initial denaturation at 95 °C for 30 s, followed by 40 cycles of 95 °C for 10 s and 60 °C for 30 s, with a default melting curve analysis program. The Novazenz ChamQ Universal SYBR qPCR Master Mix (Vazyme Biotech Co., Ltd., Nanjing, China) was employed to amplify fivefold-diluted cDNA templates. All qPCR reactions were performed in triplicate technical replicates, and three independent biological replicates were conducted for each treatment. The cotton GhActin gene was used as the internal reference gene with the following primers: *GhActin*-F (TGTCCGTCAGGCAACTCAT) and *GhActin*-R (ATCCTCCGTCTTGACCTTG). The primers used for amplification of the target gene *GhLRX7* were as follows: F (GTGCCGTCGAGTCTTGGTAA) and R (TGCTCAAGGCTCACCAGTTC). Gene-specific primers were designed using NCBI Primer-BLAST(accessed on 21 September 2022)following standard criteria (length 18–25 bp, GC content 40–60%, Tm 55–65 °C, amplicon size 80–200 bp), and their specificity was validated by a single peak in qPCR melting curve analysis, confirming the absence of non-specific amplification or primer-dimer formation. The 2^–ΔΔCT^ method [94] was applied to determine the transcript abundance of the genes of interest.

### 4.11. VIGS of GhLRX7 and Inoculation with VW

VIGS validation of the role of *GhLRX7* in cotton plant response to VW stress was performed using upland cotton cultivars MBI9626 and CCRI36. A specific 300 bp fragment of the *GhLRX7* gene was isolated from CCRI 36, a *G*. *hirsutum* cultivar, via PCR amplification with primer pairs *GhLRX7*-V-F, GTGAGTAAGGTTACCGAATTCTGAGGTTCAATGAGTTTGAA, and *GhLRX7*-V-R, CGTGAGCTCGGTACCGGATCCAGTTCTTAAGATTACCCACTTCTTCG, and then was cloned into the pYL156 VIGS vector, and the resulting construct (*TRV:GhLRX7*) was introduced into 10-day-old cotton seedlings through helper vector pYL192-mediated co-transformation. System efficacy was confirmed by the inclusion of both negative and positive controls: with the empty pYL156 vector (*TRV:00*) serving as the former, and the pYL156-*GhCLA1* construct (*TRV:GhCLA1*), known to induce a photobleaching phenotype, serving as the latter. Upon observation of the bleaching phenotype in positive controls at two weeks post-injection, transcript levels of *GhLRX7* were quantified by applying the 2^–ΔΔCT^ algorithm in qRT-PCR assays. The activated spore suspension of *V. dahliae* strain V991 was adjusted to a concentration of 1 × 10^7^ spores/mL using Czapek broth and then inoculated into the root system of each cotton seedling with 2 mL of the prepared spore suspension. Following inoculation, the plants were cultivated in a controlled environment (25 °C) to let the pathogenesis develop. At 25 DAI, phenotypes of silenced and control plants were recorded and compared, clarifying the functional role of *GhLRX7* in conferring VW resistance to the plants.

## 5. Conclusions

This genomic study delineates 89 *LRX* genes from four cotton species, categorizing them into four groups. Collinearity assessment revealed that segmental and tandem duplications were the predominant drivers of this gene family’s expansion. Expression profiling implicated LRX genes in diverse developmental processes and stress responses. Importantly, functional evidence from silencing verification of *GhLRX7* demonstrated its critical role in conferring cotton resistance to VW. Collectively, these findings provide a foundational understanding of cotton LRX proteins and suggest their potential utility in breeding crops with enhanced VW stress resilience.

## Data Availability

The original contributions presented in this study are included in the article/Supplementary Material. Further inquiries can be directed to the corresponding authors.

## Supplementary Materials

The following supporting information can be downloaded at: https://www.mdpi.com/article/10.3390/ijms27093852/s1.
