# Supplementary material for: Leucine-Rich Repeat Extension 7 Gene Confers Cotton Resistance to Verticillium Wilt
Source: Int J Mol Sci. 2026 Apr 26;27(9):3852. doi: 10.3390/ijms27093852 (PMC13164035; doi:10.3390/ijms27093852)
Supplement: Supplementary file 1 [file ijms-27-03852-s001.zip › Table S3. A whole-genome homology alignment between G. hirsutum and A. thaliana.docx]

| **Arabidopsis thaliana** | **Gossypium hirsutum** |
| --- | --- |
| AtLRX1 | GhLRX3、GhLRX5、GhLRX6、GhLRX8、 GhLRX9、GhLRX15、GhLRX18、GhLRX19、  GhLRX22、GhLRX23、GhLRX29 |
| AtLRX2 | GhLRX20 |
| AtLRX3 | GhLRX7、GhLRX10、GhLRX11、GhLRX12、 GhLRX21、GhLRX24、GhLRX25 |
| AtPEX4 | GhLRX1、GhLRX2、GhLRX4、GhLRX16、  GhLRX17、GhLRX27、GhLRX13、GhLRX26、  GhLRX14、GhLRX28 |
