# Supplementary figures and images for: Leucine-Rich Repeat Extension 7 Gene Confers Cotton Resistance to Verticillium Wilt
Source: Int J Mol Sci. 2026 Apr 26;27(9):3852. doi: 10.3390/ijms27093852 (PMC13164035; doi:10.3390/ijms27093852)

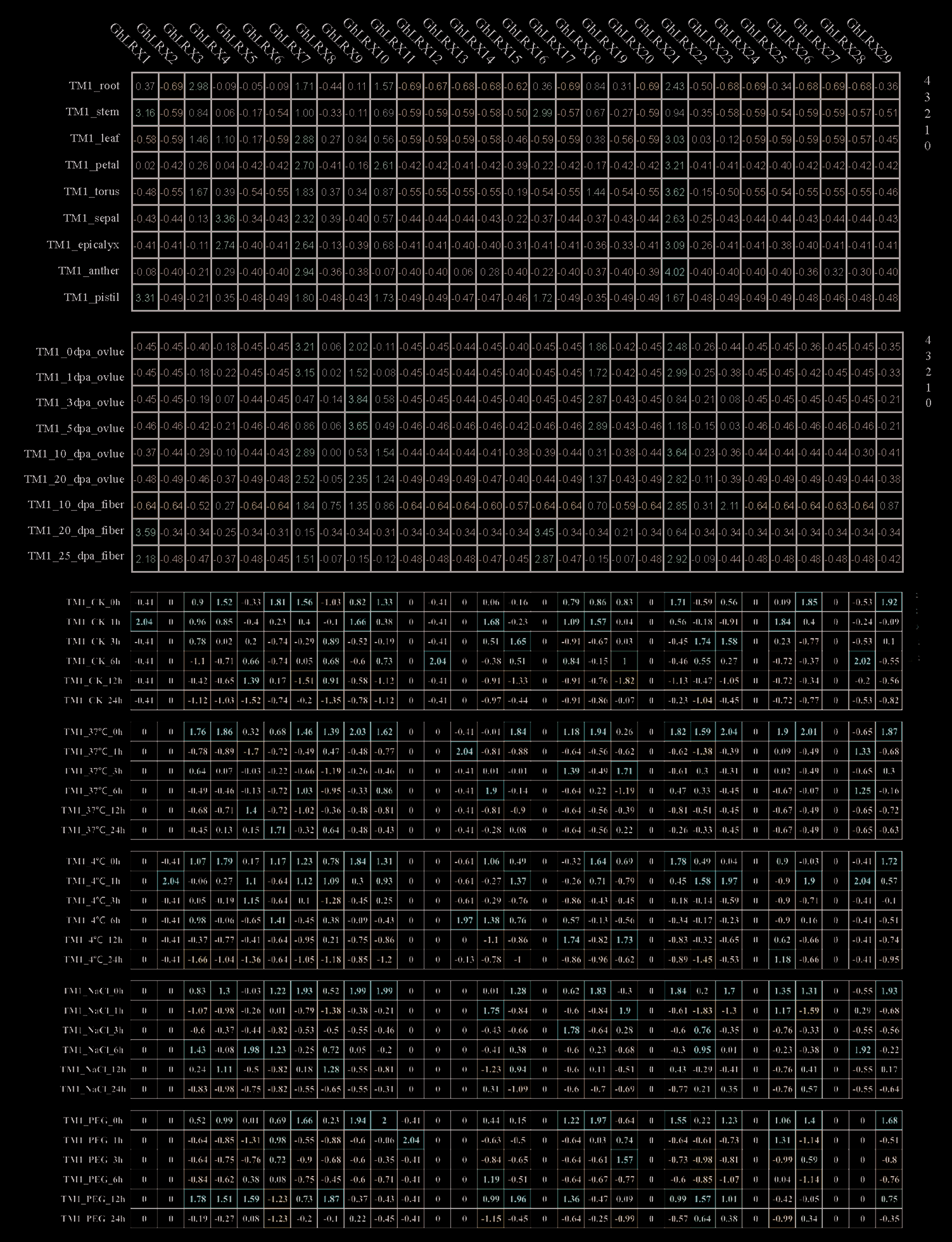

Supplement: Supplementary file 1 [file ijms-27-03852-s001.zip › Figure S2. Expression profile analysis of GhLRX genes..tif]

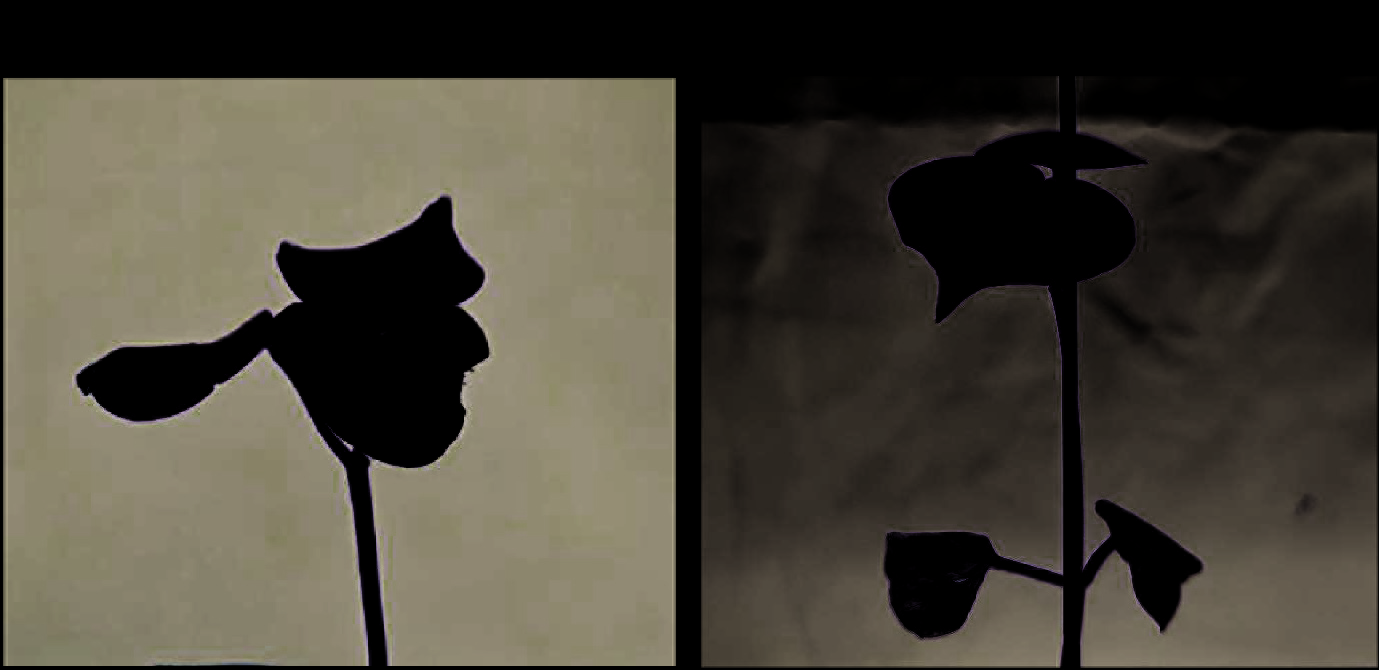

Supplement: Supplementary file 1 [file ijms-27-03852-s001.zip › Figure S3. VIGS controls (positive and negative).tif]

Motif 1

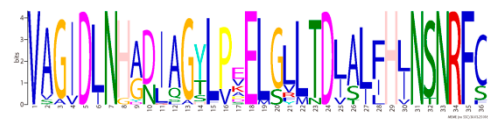

Motif 2

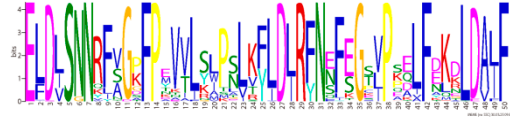

Motif 3

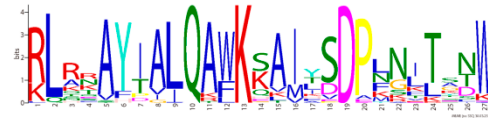

Motif 4

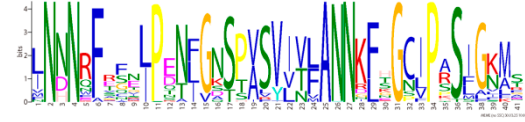

Motif 5

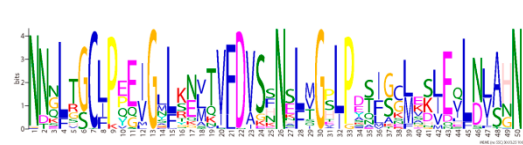

Motif 6

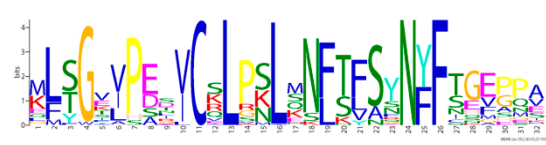

Motif 7

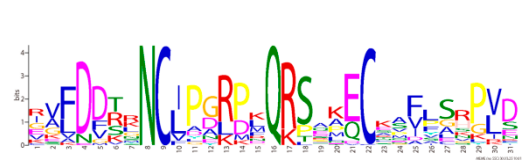

Motif 8

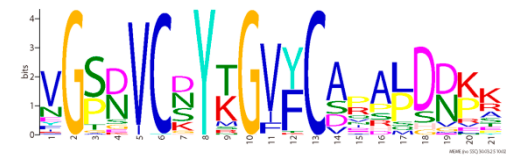

Motif 9

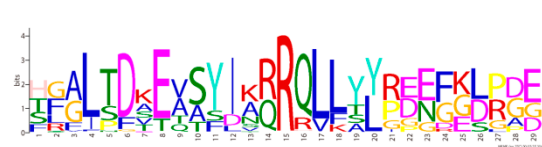

Motif 10

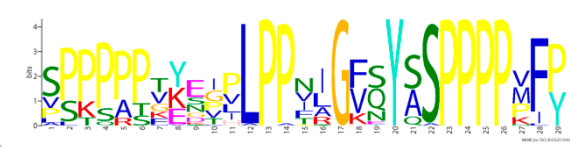

Supplement: Supplementary file 1 [file ijms-27-03852-s001.zip › Supplementary File S2. Sequence logos of conserved motifs identified in cotton LRX proteins..pdf]
